# Effects of topical corticosteroids and lidocaine on *Borrelia burgdorferi* sensu lato in mouse skin: potential impact to human clinical trials

**DOI:** 10.1038/s41598-020-67440-5

**Published:** 2020-06-29

**Authors:** Bastien Lefeuvre, Paola Cantero, Laurence Ehret-Sabatier, Cedric Lenormand, Cathy Barthel, Chrystelle Po, Nikhat Parveen, Antoine Grillon, Benoit Jaulhac, Nathalie Boulanger

**Affiliations:** 1 Fédération de Médecine Translationnelle - UR7290, Virulence bactérienne précoce-groupe Borrelia, Université de Strasbourg, 67000 Strasbourg, France; 2 Laboratoire de Spectrométrie de Masse BioOrganique, CNRS, IPHC UMR 7178, Université de Strasbourg, 67000 Strasbourg, France; 3 Clinique dermatologique, Hôpital Universitaire de Strasbourg, Strasbourg, France; 4 ICube UMR 7357, Université de Strasbourg/CNRS, Fédération de Médecine Translationnelle de Strasbourg, 67000 Strasbourg, France; 5 Microbiology, Biochemistry and Molecular Genetics, Rutgers-New Jersey Medical School, ICPH Building, 225 Warren Street, Newark, NJ 07103 USA; 6 French National Reference Center on Lyme borreliosis, Centre Hospitalier Régional Uinversitaire de Strasbourg, 67000 Strasbourg, France

**Keywords:** Diseases, Infectious diseases, Microbiology, Bacteria, Infectious-disease diagnostics

## Abstract

Lyme borreliosis is the most prevalent vector-borne disease in northern hemisphere. *Borrelia burgdorferi* sensu lato spirochetes are transmitted by *Ixodes* species ticks. During a blood meal, these spirochetes are inoculated into the skin where they multiply and often spread to various target organs: disseminated skin sites, the central nervous system, the heart and large joints. The usual diagnosis of this disease relies on serological tests. However, in patients presenting persistent clinical manifestations, this indirect diagnosis is not capable of detecting an active infection. If the serological tests are positive, it only proves that exposure of an individual to Lyme spirochetes had occurred. Although culture and quantitative PCR detect active infection, currently used tests are not sensitive enough for wide-ranging applications. Animal models have shown that *B. burgdorferi* persists in the skin. We present here our targeted proteomics results using infected mouse skin biopsies that facilitate detection of this pathogen. We have employed several novel approaches in this study. First, the effect of lidocaine*,* a local anesthetic used for human skin biopsy, on *B. burgdorferi* presence was measured. We further determined the impact of topical corticosteroids to reactivate *Borrelia* locally in the skin. This local immunosuppressive compound helps follow-up detection of spirochetes by proteomic analysis of *Borrelia* present in the skin. This approach could be developed as a novel diagnostic test for active Lyme borreliosis in patients presenting disseminated persistent infection. Although our results using topical corticosteroids in mice are highly promising for recovery of spirochetes, further optimization will be needed to translate this strategy for diagnosis of Lyme disease in patients.

## Introduction

Lyme borreliosis is caused by *Borrelia burgdorferi* sensu lato (sl) group of spirochetal bacteria that is transmitted by a hard tick belonging to the *Ixodes ricinus* complex in North America and Eurasia. After inoculation of bacteria into the skin during a blood meal, the first clinical manifestation in the majority of patients is inflammatory response in skin at the site of infection known as erythema migrans (EM). *Borreliae* often disseminate to target organs: the central nervous system, joints, heart and the distant skin^1^. In the early local stage of infection, the clinical characteristics of EM are distinctive enough to allow diagnosis and treatment immediately. If EM is not detected, the clinical manifestations of disseminated infections are quite variable based upon the infecting strain and often are less specific. Thus, microbiological demonstration of the *Borrelia* infection is important. Several methods can be used, including culture but is not routinely used because it is time consuming, expensive, requires technical expertise and exhibits a low sensitivity for diagnosis of disseminated disease manifestations^2^. Quantitative PCR (qPCR) has also been performed by some laboratories, especially for synovial fluid, where it shows up to 80% sensitivity. Sensitivity of qPCR tends to be much lower for cerebrospinal fluid samples. Finally, indirect diagnosis using two-tier serological tests is routinely used in which the first tier is usually an ELISA, followed by immunoblotting only if ELISA results are positive or equivocal^3,4^. This method has several limitations: while its sensitivity is excellent in late stages of Lyme disease, positive predictive value is diminished by a high seroprevalence in healthy individuals from endemic areas, as well as by the fact that anti-*Borrelia* antibodies could persist for the entire life of a cured Lyme borreliosis patient. A positive serological result can thus never be interpreted as a reliable demonstration of the active presence of *Borrelia* in symptomatic patients, highlighting the need for alternative methods of diagnosis of an active infection. The disseminated disease can then be treated with different antibiotics such as amoxicillin, doxycycline or ceftriaxone^5^. In fact, treatment of patients early in infection usually completely resolves symptoms of Lyme disease. However, some symptoms, primarily manifested as persistent Lyme arthritis and chronic fatigue may persist in a number of patients after completion of antibiotic treatment regimen^6^.

Mouse model is often used to investigate physiopathology of human diseases when available. Lyme disease is well investigated particularly in susceptible C3H/HeN mice where *B. burgdorferi* infection results in inflammatory carditis and arthritic manifestations^7^. This mouse model has also allowed researchers to determine association of the specific *Borrelia* strains genotypes with either spirochetes dissemination or their lack of dissemination^8,9^. The skin has also been demonstrated as a site for *B. burgdorferi* strains multiplication and persistence using this animal model^10,11^.

As the role of the skin is extensively documented now for vector-borne diseases^12–14^, we developed a new approach for diagnosis of Lyme borreliosis by using proteomics analyses of the skin biopsies^15,16^. We set up the model in Lyme-infected mouse in early and late disseminated infections to identify protein markers for detection of *B. burgdorferi* sl infection. We identified protein markers of infection, mainly OspC, flagellin and DbpA in early infections^15^ and flagellin, GAPDH, different VlsE proteins late in infection^16^. Although we obtained promising results in the mouse model, translation of this approach to humans for diagnosis was not straightforward and presented several difficulties.

To overcome problems associated with adopting our approach for patients, we more carefully compared the protocols used in the mouse model. For early diagnosis of Lyme disease in patients, we suspected that the presence of lidocaine used as local anesthetic and the presence of host blood were critical for targeted proteomics. In addition, the low level of bacteria in the mouse skin during disseminated infection did not allow the detection of *Borrelia* proteins by proteomics. Therefore, we used a local immunosuppressive drug, clobetasol, to induce *Borrelia* proliferation in situ followed by PCR quantification and identification of protein markers of *B. burgdorferi* infection. We performed additional experiments in our mouse model to better define the effect of clobetasol in the infected skin and determine its direct effect on *Borrelia* protein expression. This has prepared us for an extensive clinical trial in patients demonstrating disseminated infections in the future.

Translational research and application of our protocols developed in the mouse model to humans will require further adjustments. In this paper, we focus on the effect of lidocaine and clobetasol on *Borrelia* present in the skin in C3H/HeN mouse model to optimize conditions for a novel diagnostic test development using targeted proteomics using skin of patients afflicted with Lyme borreliosis.

## Results

### Effect of lidocaine on *Borrelia* viability in vitro

In the past, lidocaine was suggested to exhibit antimicrobial activities^17^. In fact, a recent review described antimicrobial effects of local anesthetics when collecting biological fluids for microbial studies^18^. This local anesthetic significantly inhibits the growth of bacteria; such that it can possibly also affect *Borrelia*-infected skin when used before the human biopsy specimens are collected. To evaluate this possibility, we first tested the direct effect of lidocaine in vitro on *B. afzelii*. Lethal effects of lidocaine were determined by the loss of motility and disruption of the spiral shape of *Borrelia*. It indeed demonstrated lethal effects of lidocaine at the highest concentration we used, 5 mg/ml, at day 1 and 7 (Table 1) and even its lower, 2.5 mg/ml concentration. We obtained similar data with *B. burgdorferi* ss 297 strain (data not shown). The concentration generally used in patients for skin biopsy is 10 mg/ml (0.5 ml inoculation) suggesting our need to optimize its concentration for humans that will not affect Lyme spirochetes adversely.

**Table 1:** The effect of lidocaine on *B. afzelii* strain NE4049 in vitro.

Compounds	Concentration	Day 1	Day 7
Control		++++	++++
Lidocaine (mg/ml)	5	0	0
	2.5	0	0
	1.25	++	++
	0.62	++	++
	0.31	++++	++
	0.15	++++	++++
	0.078	++++	++++
	0.039	++++	++++
	0.019	++++	++++
	0.009	++++	++++
	0.0048	++++	++++
	0.002	++++	++++
	0.001	++++	++++
	0.0006	++++	++++
Gentamicin (µg/ml)	100	0	0
	50	0	0
	25	0	0
	12.5	++	0
	6.25	++	+
	3.12	++++	++
	1.56	++++	++++
	0.78	++++	++++
	0.39	++++	++++
	0.19	++++	++++
	0.09	++++	++++
	0.048	++++	++++

*Borrelia* motility and numbers were evaluated by observation and counting using Petroff-Hausser chamber under a dark-field microscope. Gentamicin was used as a positive control.

Key used: +: detectable viable *B. afzelii* in culture by microscopy; ++: at least one spirochete/field of view; +++: at least 10 spirochetes per field of view, ++++: an average of > 100 spirochetes per field of view).

## Effect of lidocaine on *Borrelia *in vivo and its impact on proteomics data

In our mouse model, we also tested whether lidocaine can affect protein expression of *Borrelia* in the infected skin. Lidocaine affects the viability and vitality of *B. afzelii* as observed after 7 days of culture. The culture of infected skin injected with lidocaine exhibits less viable spirochetes than in control at day 7, as shown by dark field microscopy. *B. afzelii* survived in skin after lidocaine treatment because after 14 days of culture, spirochetes recovery appeared complete with spirochete numbers as numerous and motile as in the control (infected mouse without lidocaine—Table 2). Not surprisingly, no difference was observed between the two groups by qPCR since the technique detects DNA of both dead and live *Borrelia*. At day 14, the DNA detection was expressed as Ct since the skin was damaged and detection of *gapdh* and thus, relative quantification of spirochetes was not possible anymore.

**Table 2:** Effect of lidocaine injection on day 7 (peak of *Borrelia* multiplication) in the skin of C3H/HeN mice infected with *B. afzelii* NE4049.

Mouse no.	Protocol	Number and mobility of *Borrelia* in BSK-H complete medium		PCR on *Borrelia-*infected mouse skin	
		Day 7 After biopsy	Day 14 After biopsy	Day 0 (Quantification *flaB*/*gapdh* gene)	Day 7 After biopsy (Ct)
1	Infected skin at day 7	++++	++++	173	20
2		++++	++++	212	17
3		++++	++++	**252**	16
4		++++	++++	189	20
5		++++	++++	169	19
6		++++	++++	187	16
7	Infected skin at day 7 + lidocaine	++	++++	173	17
8		++	++++	182	19
9		++	++++	191	19
10		+++	++++	197	19
11		+++	++++	237	19
12		+++	++++	**271**	16

Mouse skin biopsy samples were collected at day 7 post-inoculation, and cultured for 7–14 days. To determine the spirochetes burden in skin biopsies, qPCR was also performed at day 7 without culture. After 7 days of culture, quantification of *B. afzelii* in the skin relative to host DNA was not possible due to damage to the skin in BSK-H medium, which reduced quality of mouse DNA, inhibiting *gapdh* amplification and detection. Therefore, Ct is provided for *flaB* only after 7 days of in vitro culture. Samples 3 and 12 marked in bold indicate two skin samples selected for proteomic analysis.

We also checked the effect of lidocaine on the expression of *B. afzelii* proteins to confirm our viability assay results. Interestingly, when lidocaine is injected at day 7, i.e., at the peak of *Borrelia* multiplication in the skin and samples were processed immediately (Day 0—Table 3), difference in the number of *Borrelia* proteins identified in the control and the lidocaine-treated animal was not statistically significant. Thus, 64 versus 70 bacterial proteins and more than 4,600 mouse proteins were identified. One week of culture of the skin sample allowed *B. afzelii* multiplication that improved its proteins detection. In fact, a six–sevenfold increase in the number of *Borrelia* proteins was observed after culture (Day 7—Table 3).

**Table 3:** Proteomic analysis of *B. afzelii*-infected skin.

Treatment	*fla/10*^4^* gapdh*	*fla*/*100 ng*	Number of total identified proteins	Total number of identified peptides	Number of *Borrelia* proteins	Number of *Borrelia* peptides
**Day 0 of culture**						
Without lidocaine	252	836	4,274	31,100	70	206
With lidocaine	271	838	4,685	37,300	64	186
**Day 7 of culture**						
Without lidocaine	N.d	15,200	3,140	21,733	474	2,736
With lidocaine	N.d	22,700	3,691	25,427	397	1992

At the peak of *Borrelia* multiplication in the infected skin at day 7, lidocaine was injected or not (control) before the skin biopsy. Skin was collected and directly analyzed (day 0) or cultivated for 7 days and then we selected the skin with the best qPCR count to perform proteomic analysis. For each biopsy sample, mouse and *Borrelia* proteins were identified using nanoLC-MS/MS, once for each biopsy.

Moreover, irrespective of the conditions used, we identified proteins previously detected in *Borrelia*-infected skin as infection markers in mice treated without lidocaine^15,16^, i.e. flagellin, OspC, enolase, GAPDH, lipoprotein gi|365823350, flagellar filament outer layer, elongation factor Tu, DbpA, GroEL and l-lactate dehydrogenase (Table 4).

**Table 4:** Number of identified peptides per *Borrelia* identified proteins, with or without injection of lidocaine.

	Day 0 of culture		Day 7 of culture	
	Without lidocaine	With lidocaine	Without lidocaine	With lidocaine
Flagellin	11	13	18	16
OspC	8	7	10	9
Enolase	4	2	18	16
GAPDH	13	6	24	24
Lipoprotein gi|365823350	10	8	13	11
Flagellar filament outer layer	7	7	18	18
Elongation factor Tu	5	5	23	24
DbpA	2	2	3	2
GroEL	8	8	33	33
l-lactate dehydrogenase	2	3	15	12

Skin biopsies samples were collected at the peak of multiplication, 7 days after *Borrelia* inoculation, and either directly frozen (day 0 of culture) or culture for 7 days.

Although we conducted only a single proteomic analysis for one sample each from lidocaine treated and untreated control mice, the lidocaine seems to adversely affect protein detection, since more *Borrelia* proteins were detected without lidocaine treatment after 7 days of infection in the skin biopsies (Table 3). The in vitro culture improved the number of identified peptides per *Borrelia*, in agreement with *Borrelia* multiplication observed in culture, despite lidocaine-treatment of mice (Table 4). Thus, the culture of skin-biopsy improves the health of bacteria increasing protein expression such that the efficiency of detection of *Borrelia* proteins and peptides improved more (474 and 2,736) in the skin of mice without lidocaine treatment than in the lidocaine-treated mice (397 and 1992). It is likely that culture also washes out the lidocaine reducing its impact on *B. afzelii* multiplication to prevent further exacerbation of these differences between treated and untreated samples (Boulanger-unpublished data).

We observed a similar phenomenon in the skin of patients with erythema migrans (data not shown). In vitro culture of human infected skin for several weeks allowed the detection of flagellin and OspC, which was otherwise not possible.

### Topical corticosteroid application increases localized growth of different *Borreliae* in the mouse skin 40–50 days after inoculation

**Figure 1:**
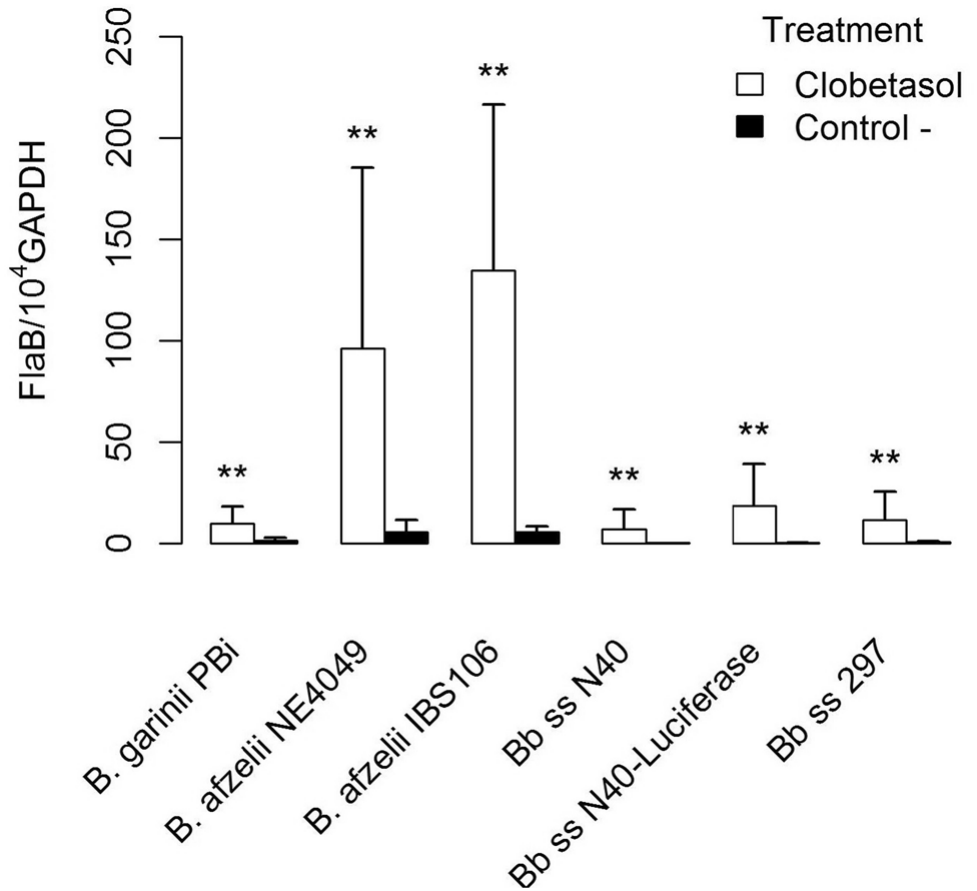
Comparison of reactivation of several *Borrelia burdgdorferi* sensu lato strains as detected by qPCR in the skin at the site of inoculation (lower back) after occurrence of disseminated infection (40–50 days post inoculation). Different groups were compared by a Mann–Whitney test and *p* values for each strain from left to right are: 0.003, 0.002, 0.008, 0.006, 0.002, and 0.001.

We first compared the reactivation of different *Borrelia* species and strains after application of clobetasol twice a day for two days at the site of inoculation (lower back of mouse). After dissemination, i.e., 40–50 days after the inoculation, *Borreliae* multiplied very well irrespective of the strain used when compared with the controls (infected but non reactivated by clobetasol) as shown by PCR quantification. We found that *B. afzelii* NE4049 (tick isolated strain) and IBS106 (human isolate) were the best *Borrelia* species strains to respond to the application of clobetasol (Fig. 1). We also tested whether local cutaneous application of clobetasol would enhance *Borrelia* multiplication at a distant anatomical site. After clobetasol application, cultures of the skin at the site of inoculation, the heart, the joint and the blood of infected mice were carried out. While a much higher quantity of *Borrelia* were recovered from the skin samples compared to the non-reactivated infected-skin, cultures of all other organs of clobetasol-treated mice remained at the same level as the untreated infected mice. Interestingly, the blood remained negative after clobetasol application. It strongly argues against any systemic effect of topical corticosteroid treatment (data not shown).

**Figure 2:**
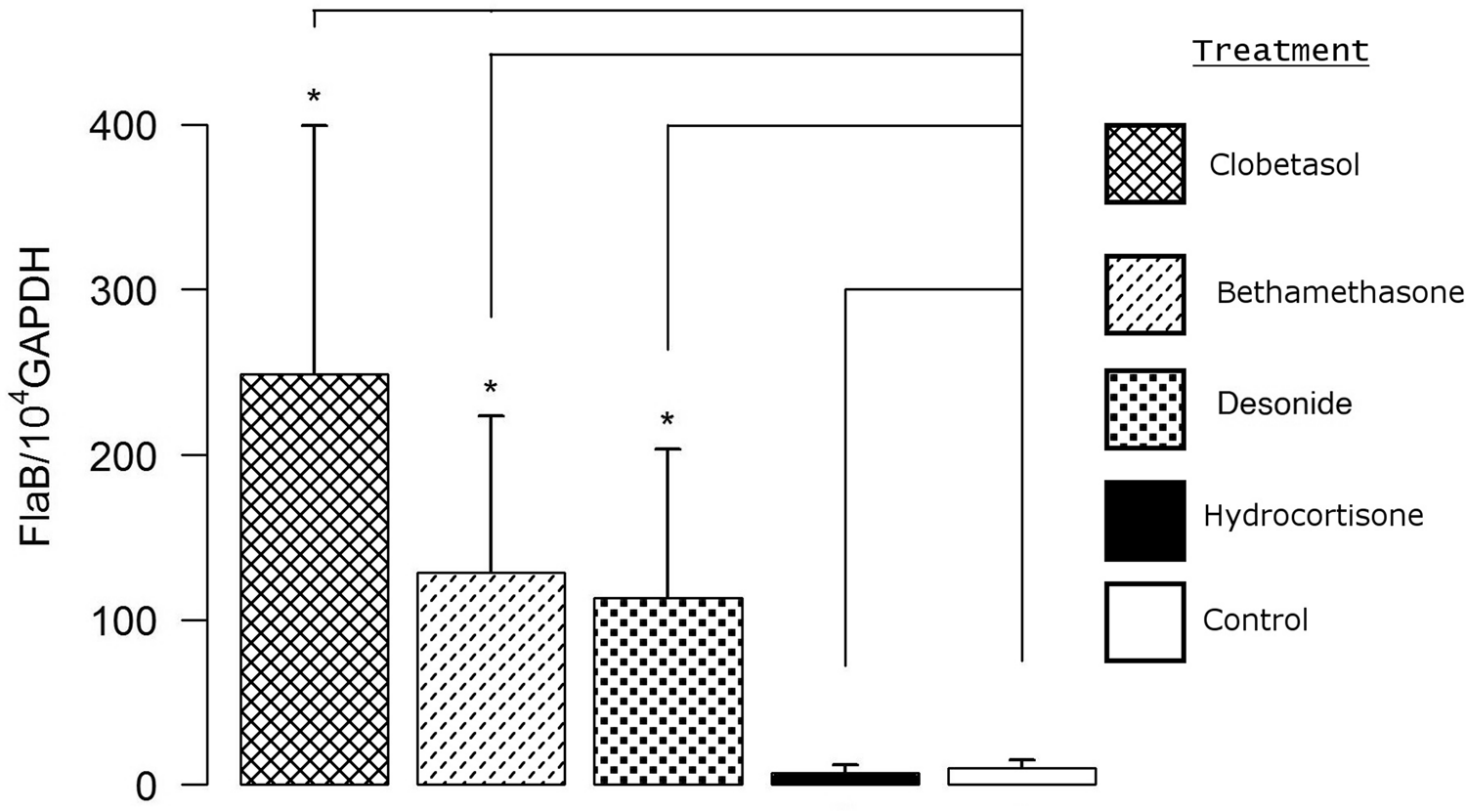
Determination of *B. afzelii* NE4049 reactivation at day 50 by qPCR, at the site of inoculation after application of topical corticoids with different levels of anti-inflammatory activities: clobetasol propionate 0.05% (very high); bethamethasone valerate 0.1% (high); desonide 0.1% (high); hydrocortisone 0.5% (low); control (non-reactivated infected skin). Different groups were compared by a Mann–Whitney test and *p* values relative to control from left to right are: 0.02, 0.02, 0.02, and 0.4.

Since *B. afzelii* multiplies very well in mouse skin, we used it to study the effect of topical corticosteroids that exhibit distinct anti-inflammatory activities^19^. Clobetasol propionate cream is considered as a corticosteroid with a very high anti-inflammatory activity, while bethametasone dipropionate and desonide cream have a high and, hydrocortisone a low anti-inflammatory activity. As shown in (Fig. 2), clobetasol was found to be most effective for bacterial reactivation. Therefore, we used it for all further experiments.

### Comparison of reactivation of the two strains of *B. afzelii* at different sites

**Figure 3:**
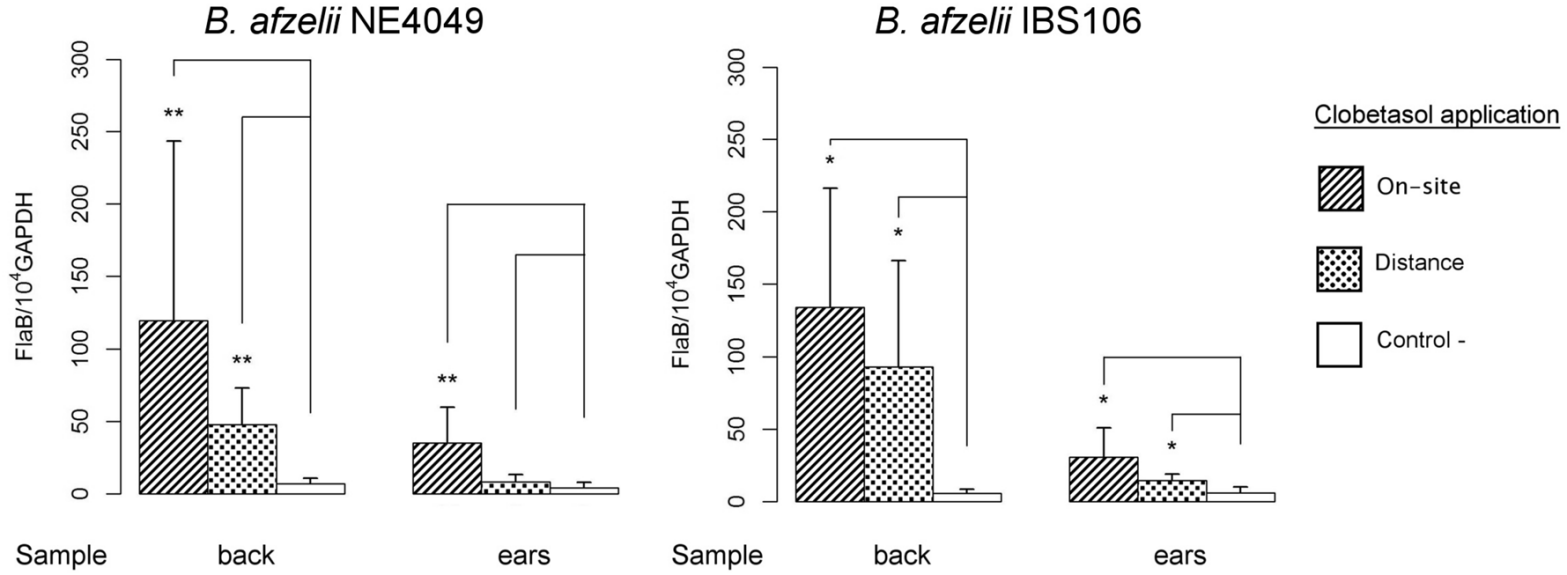
Quantification of *B. afzelii* NE4049 and IBS 106 by real-time qPCR after application of clobetasol on the back or on the ears of mice 40–50 days after *Borrelia* inoculation in the lower back of mice. Control (−) depicts infected skin collected from the back without reactivation. Different groups were compared by a Mann–Whitney test and *p* values relative to control from left to right for NE4049 are: 0.002, 0.002, 0.002, and 0.08, and for IBS 106 are: 0.01, 0.01, 0.02, and 0.01.

We examined the mechanism of *B. afzelii* reactivation more thoroughly using two strains, one isolated from a tick and another from a patient. We tested the reactivation levels and determined whether we can reactivate *Borreliae* at a site distant from the site of inoculation. When the topical corticosteroid was applied at the site of *Borrelia* inoculation (back of the mouse), *Borrelia* multiplication was detected efficiently by qPCR, primarily at the local site (Fig. 3). In the mouse model, all skin samples harbored live *Borreliae*; however, a reactivation at distance (ear) location from the site of inoculation also induced *Borrelia* multiplication in the back of mice. When the corticosteroid was applied on the ears, distant from the site of inoculation, we also detected *Borrelia* multiplication, albeit less intense than when applied at the site of inoculation. We did not observe a significant difference in the susceptibility to reactivation between the two strains of *B. afzelii*, NE4049 and IBS106 (Fig. 3).

### Reactivation of *B. burgdorferi ss* detected by determining luciferase activity in the skin

**Figure 4:**
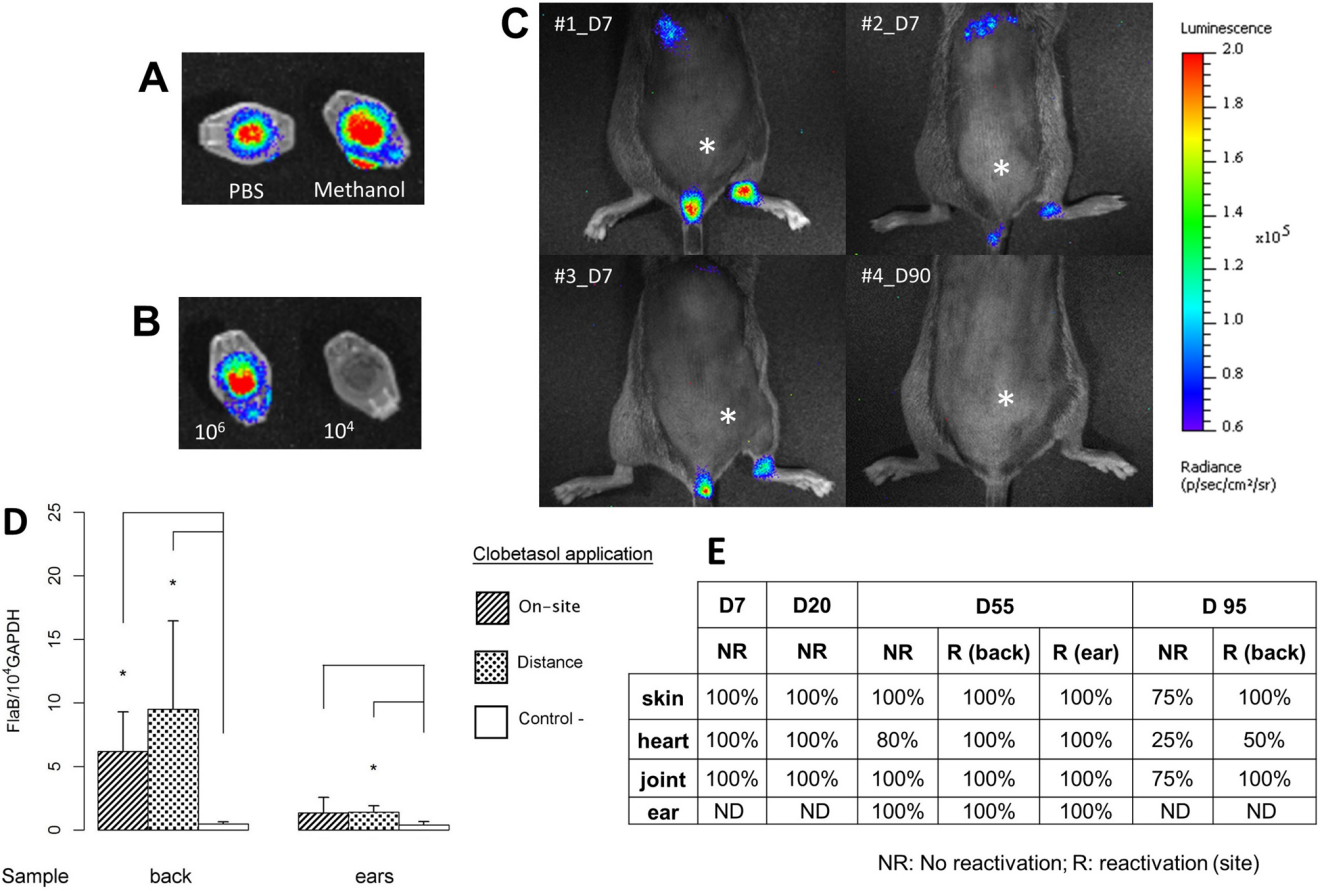
Live-imaging of luciferase-expressing *B. burgdorferi* strain N40D10/E9*. *In vitro assay was first performed to select the most suitable solvent to be used. (**A**) d-Luciferin substrate was diluted either in PBS or in methanol in Eppendorf tube to select the best solvent to detect bioluminescence. (**B**) Examination of two luciferase-expressing N40 concentrations added to the Eppendorf tubes, 10^4^ and 10^6^ spirochetes, with the substrate. In vivo assay: (**C**) Measurement of luciferase-expressing *B. burgdorferi* ss N40 strain injected in C3H/HeN mice at 7 days post-infection (natural peak of *Borrelia* multiplication-3 mice were selected) or 90 days (clobetasol reactivation—mouse#4) post-infection. The intradermal injection site of luciferase-expressing *Borrelia* is marked by an asterisk. (**D**) Quantification of luciferase-expressing N40 strain in the skin of C3H/HeN mice at day 55: the site of *Borrelia* inoculation was always at the lower back of mice, but the clobetasol reactivation was either “on site” of inoculation or “at distance”. Infected but non-reactivated mice were included as controls. The skin was collected either from the back (site of inoculation) or from the ear (distant skin from the inoculation site). Different groups were compared by a Mann–Whitney test and *p* values relative to control mice from left to right for back are: 0.01 and 0.01, and for ear are: 0.2 and 0.01. (**E**) The study was completed by culture of other tissues at different time points: heart, right joint, skin at the site of inoculation (back) and skin at a distant site (ear). The clobetasol application site is marked by an ‘R’ for reactivated mice (Day 55 and 95) or ‘NR’ for infected skin but ‘not reactivated’. “%” positive in this table was calculated using the number of mouse organ from which *Borrelia* could be recovered by culture with respect to the total number of samples cultured and examined.

To better appreciate the extent of *Borrelia* in the skin and whether the reactivation was strictly cutaneous, we used a luciferase-expressing bioluminescent *B. burgdorferi* N40/D10E9 strain^20^. We first determined the best solvent, PBS or methanol, to dissolve luciferin and defined the minimal concentration of bacteria (10^4^ and 10^6^) needed to visualize by live-imaging (Fig. 4A, B). Methanol was found to be better than PBS when used for the in vivo experiments. After injection of 10^6^ luciferase-expressing *Borrelia*, we made a kinetic analysis at 24 h, and 7, 20, 55 and 95 days post-infection. *B. burgdorferi* only reactivated with the topical corticosteroid at 55 and 95 days. Interestingly, spirochetes could only be detected by bioluminescence imaging at day 7, the usual peak of multiplication in this model^11^. The right joint, which was close to the inoculation site, was positive as well as the bladder and the heart (Fig. 4C: three representative mice are shown). Since we did not detect bioluminescence at day 20, 55 and 95 post-infection with or without reactivation, we detected *B. burgdorferi* by culture and qPCR in parallel to ensure that the spirochetes were present in the skin. As for *B. afzelii*, we confirmed the reactivation by qPCR at day 55 (Fig. 4D—other points of the kinetics not shown) and the presence of N40 in the skin and its dissemination in deep organs (heart and joint) by culture (Fig. 4E). Irrespective of the technique used and the time point selected, spirochetes were detected in the skin. However, the level of reactivation was lower for *B. burgdorferi* ss compared to *B. afzelii* that is shown in Fig. 1.

### Effect of corticosteroids on *Borrelia *in vitro

**Figure 5:**
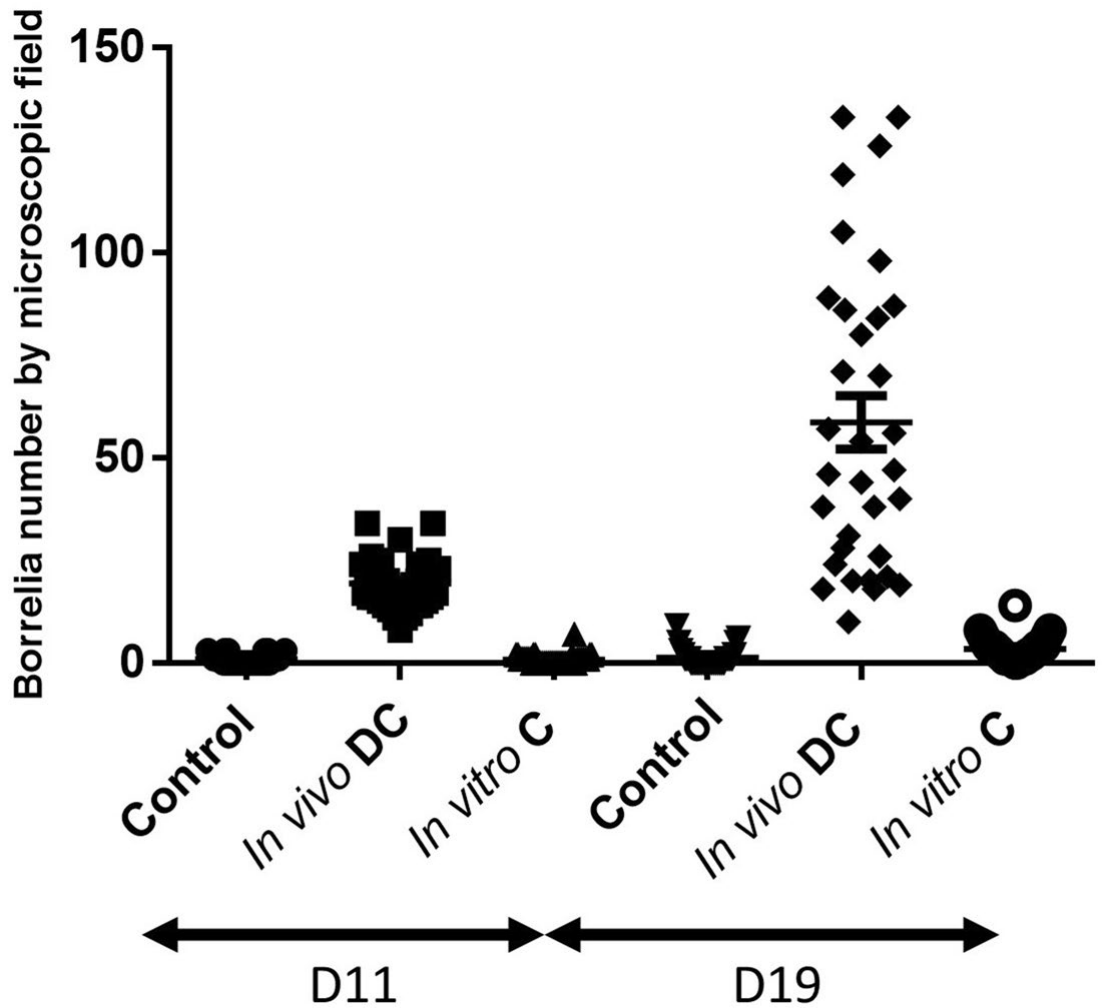
The effect of clobetasol (DC) application in vivo or incubation of *B. afzelii* NE4049-infected skin with cortisone (C) in vitro at day 40 of inoculation on the lower back of mice. Infected skins (3 biopsy punches per mouse) were incubated in BSK-H complete medium at 33 °C for 11 and 19 days and: cultured directly for control (infected but non-reactivated mouse), reactivated in vivo by skin application of clobetasol (DC) and cultured, or cultured directly with cortisone included in culture medium. *Borreliae* were counted by dark-field microscopy using a Petroff-Hausser chamber at two time points.

To test whether corticosteroids could have a direct effect on the growth of *Borreliae *in vitro and whether we need to avoid its topical application, we isolated infected skin at day 40 after *B. afzelii* inoculation, added prednisolone in vitro and measured the quantities of *Borrelia* as compared with samples of skin treated in situ with clobetasol. Mouse skins infected with *B. afzelii* were cultured for 11 and 19 days and spirochetes counted at each time point. Data in Fig. 5 shows that while skin treatment with topical corticosteroid had a strong influence on the quantities of spirochetes retrieved by culture, adding prednisolone to the culture medium had insignificant effect on bacterial multiplication. These results rule out a direct action of corticosteroids on the growth of *Borrelia,* but rather increase in spirochete numbers in the immunosuppressed skin tissue due to either increased spirochete infiltration, prevention of *Borreliae* killing by innate immune response of mice, or both. Of note, relative spirochetes quantification by qPCR was not possible using skin immersed in BSK-H complete medium since the skin was too damaged after 3 weeks to quantify mouse *gapdh* gene, but it was possible to count live spirochetes by dark field microscopy.

## Discussion

We plan to develop a novel diagnostic assay for active Lyme borreliosis employing targeted proteomics on skin biopsy specimens from patients^15^. Indeed, we suspect persistence of *Borrelia* in the skin of patients with disseminated infections as shown in mouse and dog models^7, 21^. To prepare our clinical studies, we plan to use lidocaine, a local anesthetic for local biopsy and a topical corticosteroid to reactivate *Borrelia* in the skin of patients with disseminated Lyme disease. In the present study, we show that lidocaine can affect protein detection of *B. burgdorferi* sl if the skin biopsy sample was analyzed immediately. The culture of infected skin biopsy further improved the pathogen detection. We have previously shown that topical application of corticosteroid triggers the local multiplication of *Borrelia* as detected by qPCR and culture^16^. To generate additional data on the mechanisms of corticosteroid reactivation in the skin, we examined different parameters such as the site and the level of reactivation according the class of corticosteroids used and whether the reactivation was also possible in vitro using infected skin biopsies to avoid the application of corticosteroid on the skin of patient.

The C3H/HeN mouse model represents a good system to study Lyme borreliosis and it has been studied for a long time to decipher pathology of this disease^22^. The skin was described as a site of persistence^7^ and we confirmed by different experiments that the skin could constitute a promising biological sample to develop a new diagnostic test for direct detection of *Borrelia*^11,16^. Translational projects involving proteomics have also been considered for the diagnosis of Lyme disease in patients^15^.

Diagnosis of Lyme disease in patients relies on observation of the specific clinical manifestations together with the positive serological test results. The direct detection of *Borrelia* by culture and qPCR is rarely employed and are reserved for specialized laboratories where relevant technical expertise is available^4,23^. However, serology is often unsatisfactory since in endemic areas, seroprevalence is quite high in the healthy/recovered population, so a positive result cannot be interpreted as demonstration of an active infection. In addition, some patients with late disseminated disease still suffer from persistent symptoms after completion of antibiotic treatment regimen. Several patients continue to display persistent inflammatory symptoms in absence of live bacteria^21^. Therefore, it is important to prove the absence of live *Borreliae* to ascertain the lack of active infection reducing unnecessary use of antibiotics for treatment.

We and others have previously showed that *B. burgdorferi* sl can persist in the mouse skin for several weeks, albeit at lower levels^7,16^ We have shown that a short course of clobetasol cream application can be an efficient way to stimulate the number of Lyme spirochete in the skin in mouse model, allowing increase to a level sufficient for efficient detection of *Borrelia* proteins by targeted proteomics in a reproducible manner. We conducted studies presented here using topical corticosteroids to improve recovery of spirochetes in skin biopsies and obtained data using mouse model of infection with different Lyme spirochetes. Outcomes of this study will be very useful for us to develop a diagnostic test, and after full optimization, employ it for spirochetes detection in Lyme disease patients in the future.

When a skin biopsy is part of a diagnosis in human medicine, a localized anesthetic such as lidocaine is injected before the skin biopsy. Lidocaine has been reported to exhibit antibacterial activities^17,18^. Therefore, we first investigated the effect of this anesthetic on *Borrelia*, both in vitro and in vivo. In our experiments, we demonstrated the lethal effect of lidocaine on *Borrelia* species in vitro when used at high concentrations. The injection of lidocaine at the site of spirochetes inoculation adversely affected the viability of *Borrelia* as shown by culture, and also adversely affected the proteomic sensitivity. One week of culture helped to recover live *Borreliae* allowing us to detect 7 times more proteins. Culture also likely helped in diluting the effect of lidocaine on spirochete viability. Our pilot studies with patient samples confirmed these observations (Boulanger—unpublished data).

We have previously shown that the topical corticosteroid, clobetasol can reactivate *Borrelia* in the skin in mouse model of Lyme borreliosis^16^. To better prepare the protocol for use in human patients, we further evaluated the effect of topical corticosteroid on spirochetes reactivation. First, we tested whether the three *Borrelia* species that are most pathogenic in humans reactivate similarly after clobetasol treatment. Interestingly, among all three *Borrelia* species tested, reactivation of *B. afzelii* was found to be the best after this treatment. It could be due to the fact that in the natural environment, rodents are the main reservoirs for this species^24^ and thus, is likely more adapted to mouse model than the other *Borrelia* species. In addition, *B. afzelii* is known to have a peculiar affinity for the human skin, as it is the only agent responsible for acrodermatitis chronica atrophicans, the late cutaneous manifestation of disseminated Lyme borreliosis, in which the bacteria may persist in the skin for an extended period^25^. This species is also the most frequently isolated from early cutaneous manifestations such as erythema migrans or borrelial lymphocytoma in patients from Europe^26^, but this may merely reflect the high prevalence of *B. afzelii* in ticks from this geographical area. The mechanism underlying *Borrelia* growth in murine skin following application of topical steroids is not clear. We tested different topical steroids that demonstrate differential anti-inflammatory activities. We observed a clear link between the magnitude of observed effect and the activity of the molecule tested with clobetasol proprionate being the most efficient to increase *Borrelia* recovery from mouse skin. One possible interpretation is that in mouse skin, the persistence of Lyme spirochetes is under the control of the host skin immunity. Indeed, it has been shown that *B. burgdorferi* can persist for months in the skin in absence of inflammation (M. Wooten-personal communication). Topical corticosteroids, with their local immunosuppressive effects, can likely induce a break in immune-tolerance and reactivate the persistent bacteria. Alternatively, these chemicals also create a strong vasoconstriction, which may lead to a decrease in local skin temperature. As the optimal temperature to grow Lyme spirochetes in vitro is around 33 °C and the mouse skin is approximately 37 °C, the effect of temperature may be an alternative explanation for this observation. To detect any direct effect of corticosteroids on the growth of *Borrelia*, infected skin biopsies were cultured in the presence of prednisolone. We did not observe any effect of prednisolone, likely demonstrating that corticosteroids break the tolerance of the immune system to *B. burgdorferi* sl in the skin in vivo but does not affect growth during in vitro cultivation.

We then investigated the effect of topical corticosteroids on *Borreliae* proliferation in different organ systems of mice. It has been shown that in mice^7^, like in dogs^21^, *B. burgdorferi* can be recovered following disseminated infection from every part of the skin. The application of topical steroids on the back of infected mice had a clear, although mild effect on the quantity of *Borrelia* recovered from a distant site, ears of the same animal. This finding can be attributed to the diffusion of the steroid in the skin to some extent. Importantly, this short course of treatment had no impact on spirochetes presence in other distant organs, such as the joints or the heart, and no *Borrelia* DNA was amplified from the blood, thus favoring a purely a cutaneous effect of topical corticosteroids. These data are clearly reassuring for expansion of this protocol to humans.

Using luciferase expressing *B. burgdorferi*, we only detected bacteria at day 7 post-infection. From previous experiments, we already know that this time point corresponds to an intense local multiplication of Lyme spirochetes in the mouse skin, confirming previously published data^20^. Unfortunately, the technique lacks sufficient sensitivity at later stages of infection. Therefore, we cannot as yet conclude on the extent of the reactivation after several weeks of infection. Culture and qPCR were found to be reliable methods to demonstrate the viability, persistence and amplification of *Borrelia* in the skin in all our assays.

We provide evidence that two weeks of in vitro culture can significantly improve the pathogen recovery from the skin. Up to now, most studies have focused on plasma/serum biomarkers while skin proteomics has rarely been used for diagnostics. This approach is challenging due to the putatively low recovery of pathogen components amongst a large pool of host proteins. To address this problem, the high specificity and sensitivity of targeted proteomics makes it a promising strategy. By this technique, *Mycobacterium tuberculosis* proteins were detected in human serum of infected patients^27^, and the combination with immuno-capture enabled the detection of *Yersinia pestis* proteins in dried blood spots^28^. Using skin probes as starting material, protein profiling of cutaneous leishmaniasis lesions was compared with normal skin, however, no *Leishmania* protein was identified amongst human proteins, possibly because of rather scarcity of this parasite in the skin^29^.

In the vector-borne diseases, skin has been documented as an essential interface for pathogen transmission^12,30^. More precisely, proteomic analysis could help identify new markers for persistent infection that can be used for direct diagnosis. We have successfully developed mass spectrometry-based proteomics using murine *Borrelia*-infected skin biopsies, then in human skin samples naturally infected with *Borrelia*, with quantification possible at low femtomolar range^15^. This proteomic approach is currently being evaluated for the early diagnosis of Lyme disease on a large cohort of patients (manuscript in preparation). It could then be tested for late stage of infection diagnosis since in mouse and dog models, the persistence of *B. burgdorferi* sl in the skin has been clearly demonstrated. To improve *B. burgdorferi* detection, their reactivation by topical corticosteroid can be followed by quantification using qPCR, allowing identification of the specific proteins expressed at the later stages of infection. If similar phenomenon of skin persistence exists for *Borreliae* in humans, it might constitute a new approach to identify active markers of infection in patients with disseminated infections^16^. Interestingly, persistence of cultivable *Borrelia* at the site of untreated, spontaneously cured erythema migrans has been repeatedly documented in patients with symptoms of disseminated disease, providing a strong rational to test this hypothesis^31–33^. As most patients with symptoms of late disseminated Lyme disease do not remember occurrence of a previous erythema migrans rash, a short course of topical corticosteroids on randomly chosen asymptomatic skin may be helpful to increase the sensitivity of the detection of either protein or DNA from *Borrelia* bystanders. Detection of spirochetes particularly in individuals exhibiting persistent Lyme disease manifestations will pave this new avenue for a straightforward confirmatory bacteriological diagnosis in often complex clinical situations observed in such patients.

In this paper, we emphasize the importance of filling the gap between mouse model outcomes and clinical research. Thus, we propose that protocol adjustments are necessary when a translational project is performed on humans. We clearly show that anesthetics of the lidocaine family can hamper the detection of microorganism proteins, confirming previous report^18^. We plan to develop a test for diagnosis of active Lyme borreliosis using mass spectrometry-based detection of spirochetal proteins. This new technique represents an interesting approach for the diagnosis of this disease, especially if Lyme spirochetes persist for months in the skin of humans.

## Methods

### Animals and *Borrelia* species

C3H/HeN mice (male or female) were raised at the Institute of bacteriology*.* Three different species of *Borrelia* were tested: *B. burgdorferi* ss*, B. garinii* and *B. afzelii.* Different strains belonging to these 3 main species were used for the infection experiments: *B. burgdorferi* ss strain N40D10/E9-expressing luciferase^20^, *B. burgdorferi* ss strain N40 (isolated from tick) and strain 297 (isolated from patient with neuroborreliosis), *B. afzelii* strain NE4049 (isolated from tick) and IBS 106 (isolated from a patient with erythema migrans), and *B. garinii* strain PBi (isolated from patient with neuroborreliosis). These strains were cultured in BSK-H complete medium (Sigma) at 33 °C and passaged ≤ 7 times before using them for mouse infections. Animals were infected intradermally in the lower part of the back as previously described^34^.

### Lidocaine effect in vitro and in vivo on *B. afzelii* NE4049

#### In vitro

We first tested the effect of different concentrations of lidocaine chlorhydrate without preservative (Aguettant 10 mg/ml stock solution; 5 mg/ml was the highest concentration, then twofold dilution) in vitro on a culture of *B. afzelii* NE4049. Gentamicin (100 µg/ml as highest concentration, then twofold dilution) was included as a positive control. The spirochetes were co-incubated with the different drugs in 96 well-microtiter plate for 24 h, then transferred into Eppendorf tube with 1 ml of BSK-H complete medium and incubated at 33 °C for 7 days. Observation by dark field microscopy was performed to evaluate the shape, the mobility and the density of *Borreliae* in each well at days 1 and 7.

#### In vivo

We also assessed the effect of lidocaine injection in *Borrelia*-infected skin, 7 days after *Borrelia* inoculation. Lidocaine (50 µl of a 10 mg/ml solution) was injected at the site of bacteria inoculation; allowed the anesthetic to diffuse for 30 min and then collected the skin biopsy specimens. One part was directly frozen at − 80 °C for qPCR and proteomic studies (Day 0), while the other part was cultured for 7 (Day 7) and 14 days. At day 7, qPCR and proteomics analyses were also conducted to measure the effect of culture on the expression of *Borrelia* proteins, and their detection.

### Proteomics

Mouse skin biopsies (15 mg) were manually extracted by 600 µL of Laemmli sample buffer in a 1 ml potter tissue grinder (Berktree, Cary, NC). Proteins (50 µg) were resolved by 12% SDS-PAGE^15^. Gel bands (10 ± 1 bands) of 2 mm were excised manually. Pre-digestion, digestion and nano-Liquid Chromatography-Mass Spectrometry/Mass Spectrometry (nanoLC-MS/MS) analyses were carried out as described previously^16^, once for each sample. Mass data collected during nano-LC–MS/MS were converted into “.mgf” files with MSConvert software (Proteowizard, version 3.0.6090), and interpreted using Mascot (Matrix Science, version 2.5.1). Searches were performed against an in house generated protein database composed of *Borrelia* and mouse protein sequences extracted from NCBInr and UniProtKB-SwissProt, respectively. For bacteria, four different reference databases were used depending on the strains analyzed: *B. burgorferi* ss B31 (1,758 entries at May 07, 2013), *B. burgdorferi* ss N40 (1,480 entries at January 30, 2015), *B. afzelii* PKo (2,186 entries at October 16, 2014), and *B. garinii (B. bavariensis)* PBi (1,720 entries at August 30, 2013). The Mascot results were independently loaded into Proline software (Proline Studio Release, version 1.6). All spectra leading to an identification exceeding a minimum set threshold (Mascot Ion Score > 25, peptide length > 7 amino acids, pretty rank = 1) were considered. Resulting spectra were then filtered to obtain a protein false discovery rate of less than 1%.

### Reactivation of *Borrelia* after dissemination by topical corticosteroid application

#### In situ reactivation

C3H/HeN mice (3–4 weeks old) were infected with different *Borrelia* species and strains via needle inoculation. Mice were inoculated intradermally in the dorsal thoracic area with 10^3^ spirochetes suspended in 100 μL of BSK-H. Two to 3 weeks after bacterial inoculation, the presence of *Borrelia*-specific IgG antibodies in the mice sera was determined by ELISA (data not shown). Mice were sacrificed by cervical dislocation and different organs and the blood were collected for *Borrelia* culture and/or qPCR. After bacterial dissemination, spirochetes were reactivated in mouse skin between days 40 to 90 post-infection by applying topical corticosteroid at the site of inoculation (back) or at distant site (ear), twice a day for 2 days. Non-reactivated infected mice were used as negative controls (absence of *Borrelia* reactivation).

### Comparison of *Borrelia* reactivation after application of different dermocorticoids

Topical corticosteroids with different anti-inflammatory activities^19^ have been tested in vivo: ~ 10 mg of 0.05% clobetasol propionate (very high) or 0.1% desonide (high) or 0.1% bethamethasone valerate (high) or 0.5% hydrocortisone cream were applied for one minute by massage to the dorsal thoracic area of the *B. afzelii* NE4049-infected mice, twice a day for 2 days. Quantification and *Borrelia* culture by qPCR were performed on skin biopsies collected at the site of cream application.

### Comparison of *B. afzelii* reactivation in situ or distant site

In *B. afzelii*-infected mice, two sites of clobetasol-reactivation were compared: the site of inoculation in the back and a distant site, the ear. After euthanasia, different organs were collected to detect *Borrelia* presence by culture and/or by qPCR.

### Reactivation of *Borrelia burgdorferi* ss N40-luciferase in the skin of infected mice

In order to better measure the extent of the reactivation of *Borrelia* in the skin, we carried out experiments of imaging using *Borrelia*-luciferase using codon-optimized luciferase expressing N40 D10/E9 strain^20^.

#### In vitro imaging

To validate the expression of luciferase in *B. burgdorferi* ss for in vivo imaging, first 10^4^ and 10^6^ spirochetes were treated with 50 µL of d-Luciferin sodium salt diluted in PBS or in methanol (10 or 50 mg/mL, Sigma-Aldricht) in Eppendorf tubes. Luminescence was estimated using IVIS Lumina X5 imaging system (Perkin Elmer, Massachusetts, USA) with 5 min exposures. These were the same settings used for in vivo imaging experiments (see below).

#### In vivo imaging

C3H/HeN mice were observed at different days after inoculation of *luc*-expressing *B. burgdorferi* N40 (10^6^ spirochetes injected: 3 mice observed at day 7 after infection (day 7) and 2 mice at day 90 (day 90) after infection. Before imaging on day 90, topical corticosteroid was applied on mouse skin for 2 days. Each animal was anesthetized in oxygen-rich induction chamber with 2% isoflurane and then, injected intraperitoneally with 100 µL of d-luciferin in methanol (XenoLight d-Luciferin, Perkin Elmer). Mouse was installed in ventral position in the chamber of the IVIS Lumina X5 system (Perkin Elmer). Anesthesia was maintained during the entire imaging process and animal body temperature was regulated using a digitally thermostatic bed integrated within the IVIS system. The series of luminescence images was acquired during first 40 min after d-Luciferin injection. Data acquisition and analysis were performed by using the Living Image 4.5.5 software (Perkin Elmer). Luminescence was expressed in photons/s/cm2/sr and normalized with respect to imaging time, area imaged, and the distance between the light source (i.e., the mouse) and the charge-coupled device camera.

After each imaging session, mice were euthanized and *B. burgdorferi* infected-skin (site of inoculation on the back), heart, distant skin (ear) and joint were cultured to recover live spirochetes and quantified by PCR.

#### In vitro versus in vivo reactivation with cortisone

*B. afzelii*-infected skin was collected at around day 40, after either two days of in vivo topical application with clobetasol or by adding in vitro dissolved prednisolone (20 mg) to the culture of infected skin^35^. Culturing of infected skin without reactivation served as control. The culture of infected skin biopsy punch (6 mm) was followed for 11 and 19 days observation by dark field microscopy and spirochetes counted, and quantified by qPCR.

### Culture and qPCR of *B. burgdorferi* sensu lato from mouse organs or blood

Mice were sacrificed by cervical dislocation at day 3 following topical corticosteroid treatment. One-cm^2^ area of dorsal thoracic skin, and an ear, heart and joint were removed for qPCR and culture. Blood was also collected for in vitro culture. For detection of *B. burgdorferi* sl by culture, different mouse organs were dissected aseptically. Collected organs and blood (3 drops) were placed in 6 ml of BSK-H complete medium containing 30 μg of rifampicin (BioRad). Culture tubes were incubated at 33 °C and examined weekly for the presence of spirochetes by dark-field microscopy as described previously^34^. For each organ and blood sample, tissue materials were divided into two parts: the first part was tested for live spirochetes presence following in vitro culture. If the culture remained negative after incubation, the second part was tested for DNA using qPCR. For all skin samples, culture and/or qPCR were performed.

### Estimation of *Borrelia* load in mouse skin by qPCR

Mouse tissue samples were tested for the presence of *B. burgdorferi* sl using qPCR that targeted the *flaB* gene as described previously^36^. DNA was extracted from the skin of individual mice on a MagNA Pure equipment. Quantification of the *B. burgdorferi*-specific *flaB* gene was performed on a LightCycler system (Roche Diagnostics). Quantification of the mouse-specific *gapdh* gene was performed on an ABI Prism 7,500 instrument (Applied Biosystem), using a commercial kit (TaqMan rodent GADPH control reagent; Applied Biosystem). The number of *B. burgdorferi* sl spirochetes in tissue samples was standardized per 10^4^ mouse *gapdh* gene copies^11^.

### Statistical analyses

For different kinetic analyses, between 5 and 20 mice were used for each time point of the protocol. For proteomic analyses, for different infection protocols, between 10 and 22 mice were inoculated. We selected the mouse skin samples with the highest PCR quantification to maximize protein detection. Data were analyzed with R core team version 3.6.3 (https://www.r-project.org/index.html) to compute the mean with the standard deviation (SD) and generate bar plots. Groups were compared by a Mann–Whitney test with a Benjamini Hochberg correction when multiple tests were performed. Significant *p* value is reported as follow: “*” for *p* < 0.05 and “**” for *p* < 0.01.

### Ethics statement

The protocols carried out in this study were approved by the Comité Régional d’Ethique en Matière d’Expérimentation Animale de Strasbourg (CREMEAS—Committee on the Ethics of Animal Experiments of the University of Strasbourg). Name of the ethics statement is /No. CREMEAS 2015062210282757 and APAFIS # 9317. The protocols performed on animals follow the European guidelines: “directive 2010/63/EU” under the animal facilities #: d67-482-34.
